# Comparative pharmacokinetics and bioequivalence of 145-mg fenofibrate formulations in healthy Korean participants

**DOI:** 10.1007/s00210-025-04086-y

**Published:** 2025-04-30

**Authors:** Sujong Lee, Byungwook Kim, SeungHwan Lee, Seung-Hyun Kang, Kyung-Sang Yu

**Affiliations:** 1 Department of Clinical Pharmacology and Therapeutics, Seoul National University College of Medicine and Hospital, Seoul, Republic of Korea; 2 Clinical Research Center, H-Plus Yangji Hospital, Seoul, Republic of Korea

**Keywords:** Fenofibrate, Bioequivalence, Dyslipidemia, Pharmacokinetics, Safety

## Abstract

Fenofibrate is a serum lipid-modifying agent that is commonly used to treat dyslipidemia. This study aimed to compare the pharmacokinetics (PKs) and establish bioequivalence of two 145-mg fenofibrate formulations, AD-104 (test) and TRICOR® (reference). This randomized, open-label, two-sequence, two-period crossover study was conducted in healthy Korean participants. Forty participants were enrolled and received either the test or reference formulation during each period, with a 14-day washout between doses. Blood samples were collected pre-dose and up to 72 h post-dose. PK parameters were assessed using non-compartmental analysis with Phoenix WinNonlin®. Bioequivalence was determined if the 90% confidence intervals (CIs) for the geometric mean ratios (GMRs) of the maximum plasma concentration (*C*_max_) and the area under the concentration–time curve from time zero to the last measurable plasma concentration (AUC_last_) were within the bioequivalence limits of 0.80 to 1.25. Thirty-eight participants completed the study and were included in the PK analysis. The GMR and 90% CIs for the *C*_max_ and AUC_last_ of the test compared to the reference formulation were 0.8643 (0.8283–0.9019) and 0.9930 (0.9631–1.0239), respectively, both within the bioequivalence limits. No serious adverse events were reported during the study. This study demonstrates that the test formulation is bioequivalent to the reference formulation in healthy Korean participants. Both formulations were safe and well-tolerated; therefore, AD-104 is expected to benefit Korean patients with dyslipidemia. Clinical Research Information Service No. is as follows: KCT0009332 (April 12, 2024).

**Supplementary Information:**

The online version contains supplementary material available at 10.1007/s00210-025-04086-y.

## Introduction

Dyslipidemia is an imbalance in blood lipid levels, including low-density lipoprotein cholesterol (LDL-C), high-density lipoprotein cholesterol (HDL-C), and triglycerides (TG) (Grundy et al. 2002). It represents a major risk factor for cardiovascular disease (CVD), which is the leading cause of death worldwide, contributing to an estimated 17.9 million deaths (World Health Organization 2021). The management of dyslipidemia through lifestyle changes or pharmacotherapy is recommended based on the risk of CVD (Catapano et al. 2016).

Statins are currently the first-line pharmacotherapy for dyslipidemia, supported by substantial evidence demonstrating their efficacy in reducing the risk of CVD by lowering LDL-C levels (Collaborators 2013; Grundy et al. 2019). However, reducing LDL-C levels alone may not be sufficient to fully mitigate the risk of CVD. Therefore, it is recommended that non-HDL-C levels, including TG levels, be managed to more effectively reduce the risk of CVD (Ginsberg et al. 2010; Jones et al. 2009).

Fenofibrate, a fibric acid derivative, effectively lowers elevated LDL-C, total cholesterol, and TG levels while increasing HDL-C levels in patients with dyslipidemia (Brown 1988). Fenofibrate activates peroxisome proliferator-activated receptor alpha (PPAR-α) to enhance lipolysis and promotes the elimination of TG-rich particles from plasma by activating lipoprotein lipase and suppressing apoprotein C-III production (Staels et al. 1998).

TRICOR® (Abbott Laboratories), a 145-mg fenofibrate tablet, was approved by the U.S. Food and Drug Administration (FDA) in 2004 (U.S. Food and Drug Administration 2004). It is a serum lipid-modifying agent used for treating dyslipidemia, including primary hypercholesterolemia, mixed dyslipidemia, and hypertriglyceridemia. Following oral administration, fenofibrate is rapidly hydrolyzed to its active metabolite, fenofibric acid, which has a terminal elimination half-life (*t*_1/2_) of approximately 20 h, allowing for once-daily dosing. Approximately 60% of radiolabeled fenofibrate is excreted renally as fenofibric acid and its glucuronate conjugate, while the remaining 25% is excreted via feces (U.S. Food and Drug Administration 2018). Systemic exposure to fenofibric acid is not significantly different when a single 145-mg dose of fenofibrate is administered under fasting versus fed condition, indicating that TRICOR can be administered without regard to meals. However, the time of maximum observed concentration (*T*_max_) values were prolonged from 2.33 h under fasting condition to 4.27 h under fed conditions (U.S. Food and Drug Administration, Center for Drug Evaluation and Research 2004).

AD-104, a generic version of fenofibrate developed by Addpharma Inc. in Korea, is similarly anticipated to be administered without regard to meals; however, it requires pharmacokinetic (PK) and safety data to establish its bioequivalence with TRICOR. Therefore, this study aimed to compare the PKs and evaluate the bioequivalence of two 145-mg fenofibrate formulations, AD-104 (test) and TRICOR (reference).

## Method

The study protocol was approved by the Ministry of Food and Drug Safety (MFDS) and Institutional Review Board (IRB) of H Plus Yangji Hospital. This study was conducted in compliance with the Declaration of Helsinki and the Korean Good Clinical Practice (KGCP) guidelines. Written informed consent was obtained from all participants before screening. This study was registered with the Clinical Research Information Service (CRIS No.: KCT0009332).

### Participants

Healthy Korean participants aged 19 years and older with a body mass index between 18.0 and 30.0 kg/m^2^ were recruited. The participants were screened based on their medical history, physical examination, vital signs, and clinical laboratory tests (hematology, blood chemistry, urinalysis, and serology). Exclusion criteria included participation in another clinical trial within 6 months prior to the first dose of the investigational product (IP), blood aspartate transaminase (AST), and alanine aminotransferase (ALT) levels exceeding twice the normal upper limits, or known fenofibrate hypersensitivity. A detailed list of inclusion and exclusion criteria is provided in the Supplementary Information.

### Study design

This was a randomized, open-label, two-sequence, two-period crossover study conducted to compare the PKs and safety of the test (AD-104, Addpharma, Inc., Korea) and reference fenofibrate formulations (TRICOR tablets, AbbVie Inc.). Forty participants were randomly assigned to one of two sequences (reference to test formulation or vice versa) and received their IPs with 150 mL of water.

The participants fasted for at least 10 h before IP administration and were not allowed to consume water for 1 h before and after IP administration, except for the water needed for IP administration. A meal was provided 4 h after IP administration. A 14-day washout period was included to prevent carry-over effects between doses.

Blood samples for PK analysis were collected at pre-dose and 0.5, 1, 1.5, 2, 2.5, 3, 3.5, 4, 4.5, 5, 6, 7, 8, 12, 24, 48, and 72 h post-dose. Samples were centrifuged at 3000 rpm for 10 min at 4 °C, and the plasma aliquots were stored at − 70 °C until analysis. Stability studies confirmed that fenofibric acid stored at − 70 °C remained stable throughout the entire study period, specifically up to 76 days.

### Bioanalytical methods

Plasma concentrations of fenofibric acid were quantified using a validated liquid chromatography-tandem mass spectrometry (LC–MS/MS) method. Analyses were conducted using a Waters ACQUITY UPLC™ (Waters, Milford, MA, USA) coupled with a Waters XevoTM TQ MS mass spectrometer (Waters, Wilmslow, UK) in positive ion electrospray ionization mode. Chromatographic separation was performed using a C18 column (2.1 mm ID × 50 mm L, 1.7 μm particle size; Waters ACQUITY UPLC® BEH C18; Waters, Milford, MA, USA) maintained at 30 ± 5 °C. The mobile phase consisted of water containing 0.1% formic acid and acetonitrile containing 0.1% formic acid, delivered at a flow rate of 0.3 mL/min. Multiple reaction monitoring were used for mass spectrometric detection, with mass transitions of 319.10 → 233.10 m/z for fenofibric acid and 325.10 → 233.10 m/z for the internal standard, fenofibric aicd-d_6_. Calibration curves demonstrated correlation coefficients exceeding 0.9992 for all plasma sample batches within a validated concentration range of 50.0–20000 ng/L (Supplementary Table 1). Full details of the validation results for plasma fenofibric acid are provided in the Supporting Information.

A total of 1404 plasma samples were analyzed across ten analytical batches, including incurred sample reanalysis (ISR) to assess assay reproducibility. Two ISR samples exceeded acceptance criteria due to sample-switching errors and were reanalyzed twice within the respective batches. Following reanalysis, all ISR results and batch analyses met regulatory bioanalytical validation criteria, confirming assay robustness.

### Pharmacokinetic analysis

The following PK parameters were calculated using non-compartmental analysis with Phoenix WinNonlin® (version 8.4; Pharsight, Sunnyvale, CA, USA): the area under the concentration–time curve from time zero to the last measurable plasma concentration (AUC_last_), the AUC from zero to infinity (AUC_inf_), and the *t*_1/2_ of fenofibric acid. The AUC was determined using the linear trapezoidal linear interpolation method. The *t*_1/2_ was calculated as ln(2)/terminal elimination rate (λz). The *T*_max_ and maximum observed plasma concentration (*C*_max_) of fenofibric acid were determined using the observed plasma concentration–time profiles. Participants who completed the PK sampling were included in the PK analysis.

### Safety assessment

Safety and tolerability were assessed by monitoring adverse events (AEs), physical examinations, vital signs (blood pressure, pulse rate, and body temperature), and clinical laboratory tests (hematology, blood chemistry, and urinalysis). All AEs were classified according to treatment group and coded by system organ classes and preferred terms using the Medical Dictionary for Regulatory Activities (MedDRA® version 21.1). AEs were summarized by treatment group based on severity (mild, moderate, or severe) and their relationship with the IP. Participants who received at least one IP dose were included in the safety assessment.

### Statistical analysis

The sample size calculation determined that 40 participants were needed to evaluate bioequivalence based on an intra-subject variability (ISV) of 27.2% for the AUC_last_ of fenofibric acid at a significance level of 0.05, and a power of 80% (Chow and Wang 2001; U.S. Food and Drug Administration, Center for Drug Evaluation and Research 2009). Accounting for an anticipated 20% dropout rate, a total of forty participants were enrolled.

SAS software (version 9.4; SAS Institute Inc., Cary, NC, USA) was used for statistical analysis. Descriptive statistics were reported, including the mean ± standard deviation (SD) for continuous data and frequencies and percentages for categorical data. An analysis of variance (ANOVA) was performed on the log-transformed PK parameters using a linear mixed-effects model with period, sequence, and treatment as fixed effects and participants nested within the sequence as a random effect to compare the PKs of the two fenofibrate formulations. Bioequivalence was confirmed if the 90% confidence intervals (CIs) for the geometric mean ratios (GMRs) of *C*_max_ and AUC_last_ fell within the bioequivalence limits of 0.80 to 1.25 (Ministry of Food and Drug Safety (KR) 2023).

## Results

### Study dispositions and demographics

**Fig. 1:**
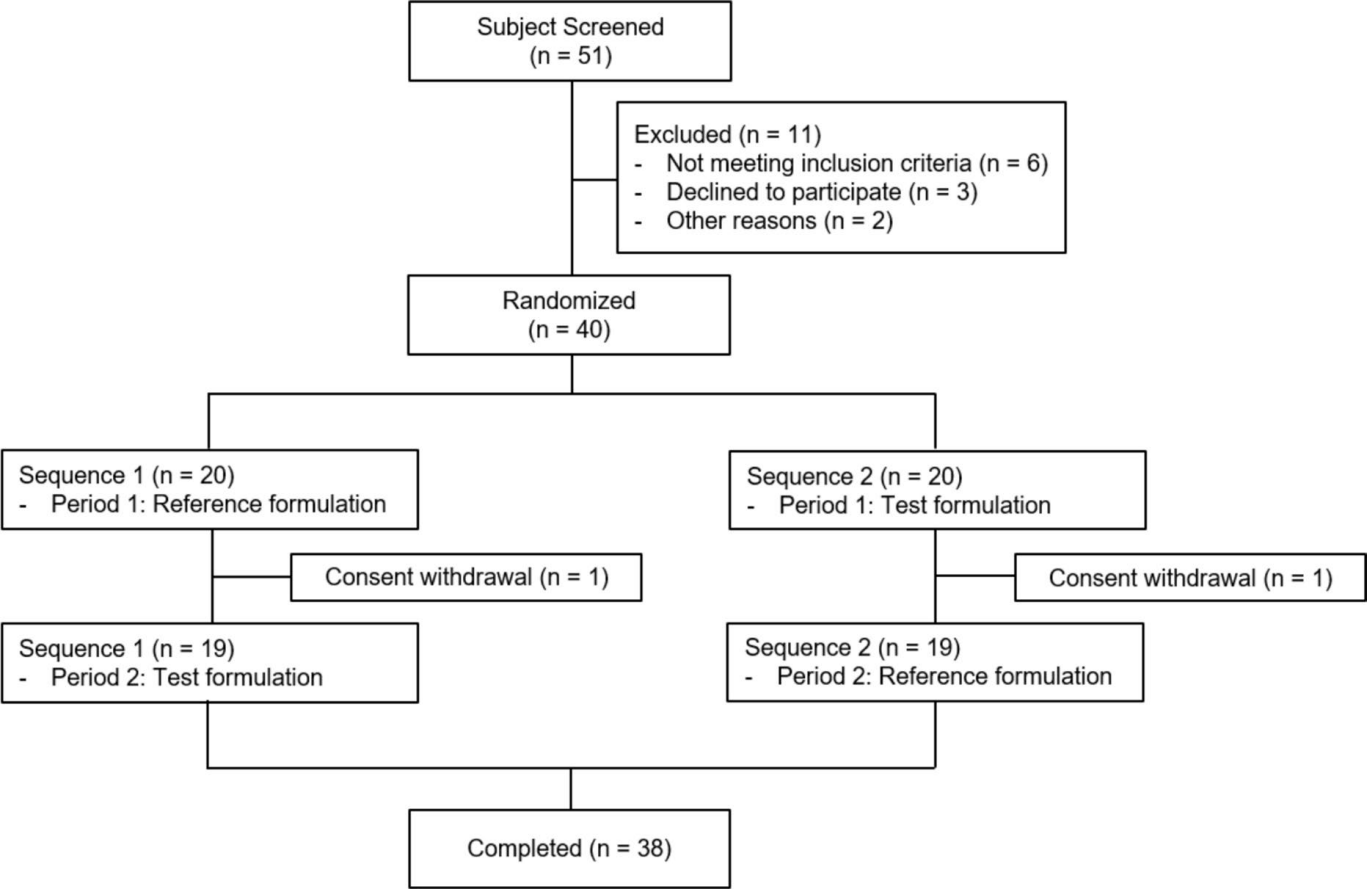
Participant flow

A total of 51 participants were screened, 11 of whom were excluded for various reasons (Fig. 1). Forty male participants were randomized, and two withdrew their consent after the first study period. All 40 participants were included in the safety assessment, and 38 who completed the study were included in the PK analysis. The mean ± SD for age, weight, height, and BMI of the 40 enrolled participants were 27.88 ± 5.61 years, 71.47 ± 9.90 kg, 174.77 ± 6.02 cm, and 23.37 ± 2.88 kg/m^2^, respectively. Demographic characteristics, including age, weight, height, and BMI, were similar between the two groups (Table 1).

**Table 1:** Participant demographics

Variables	Sequence 1 (*n* = 20)	Sequence 2 (*n* = 20)	Total (*n* = 40)
Age (year)	27.75 ± 5.14	28.00 ± 6.18	27.88 ± 5.61
Height (cm)	175.01 ± 6.26	174.53 ± 5.93	174.77 ± 6.02
Weight (kg)	69.30 ± 11.32	73.65 ± 7.93	71.47 ± 9.90
BMI (kg/m^2^)	22.54 ± 3.07	24.20 ± 2.48	23.37 ± 2.88

Data are presented as arithmetic mean ± standard deviation. BMI was calculated as weight (kg)/height^2^ (m^2^)

Sequence 1 was reference to test formulation. Sequence 2 was test to reference formulation

*BMI* body mass index

### Pharmacokinetics

**Fig. 2:**
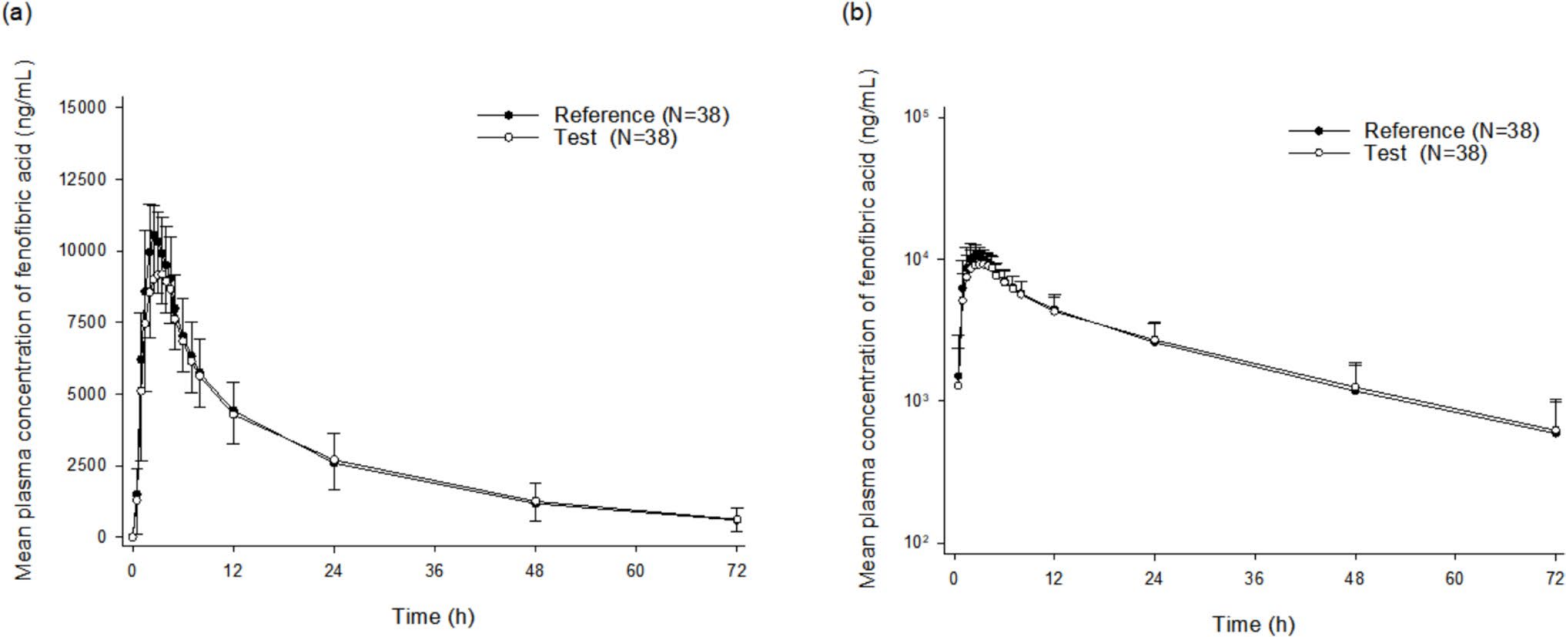
Mean plasma concentration–time profiles of fenofibric acid after a single oral administration of two 145-mg formulations presented on **a** linear and **b** log scales. The error bars indicate the standard deviations

The mean plasma concentration–time profiles of fenofibric acid are shown in Fig. 2, and the PK parameters are summarized in Table 2. The PK profiles of the test formulation after a single oral dose were comparable to those of the reference formulation (Fig. 2). Both formulations showed similar absorption and elimination profiles for fenofibric acid, with a median *T*_max_ of 2.0 to 2.5 h and a mean *t*_1/2_ of approximately 20 h. The GMRs and 90% CIs for the *C*_max_ and AUC_last_ of the test formulation relative to the reference formulation were 0.8643 (0.8283–0.9019) and 0.9930 (0.9631–1.0239), respectively, both within the bioequivalence limits of 0.80 to 1.25. Changes in *C*_max_ and AUC_last_ values after the administration of both formulations to each participant are shown in Supplementary Fig. 1. For most participants, both *C*_max_ and AUC_last_ remained within narrow ranges across the test and reference formulations.

**Table 2:** Pharmacokinetic parameters of fenofibric acid after a single oral administration of two 145- mg fenofibrate formulations

Parameters	Test (*n* = 38)	Reference (*n* = 38)	Geometric mean ratio (90% CI)
*T*_max_ (h)	2.500 [1.500–4.500]	2.000 [1.000–4.500]	-
*C*_max_ (ng/mL)	9930.000 ± 2291.545	11,371.316 ± 2088.803	0.8643 (0.8283–0.9019)
AUC_last_ (h·ng/mL)	186,768.845 ± 50,556.129	188,564.247 ± 52,802.578	0.9930 (0.9631–1.0239)
AUC_inf_ (h·ng/mL)	209,709.410 ± 69,427.270	209,194.970 ± 69,292.618	1.0041 (0.9696–1.0399)
*t*_1/2_ (h)	21.653 ± 6.668	21.264 ± 4.976	1.0046 (0.9617–1.0494)

Data are presented as arithmetic mean ± standard deviation, except for *T*_max_, which is presented as the median [minimum–maximum]. The symbol “-” indicates that the data are not presented. The geometric mean ratios calculations of the test to reference formulation are based on log-transformed data

*CI* confidence interval, *T*_*max*_ time of maximum observed concentration, *C*_*max*_ maximum observed concentration, *AUC*_*last*_ area under the concentration–time curve from time zero to the last measurable plasma concentration, *AUC*_*inf*_ area under the concentration–time curve from time zero to infinity, *t*_*1/2*_ terminal elimination half-life

### Safety

All 40 participants who received at least one dose of the IP were included in the safety assessment. Eight treatment-emergent AEs (TEAEs) were reported in six participants (15%), all related to the IP and classified as adverse drug reactions (ADRs). In the test formulation group, five ADRs (white blood cells urine positive, blood glucose increased, hemoglobin decreased, and red blood cells urine positive) were observed in four participants (10.3%). In the reference formulation group, three ADRs (lipase increased, neutrophil percentage increased, and nausea) were observed in two participants (8.3%) (Supplementary Table 1). All AEs were mild, except for one moderate AE (nausea) in a participant who received the reference formulation. No serious AEs occurred during the study period.

## Discussion

This study was conducted to compare the PKs and determine the bioequivalence of two 145-mg fenofibrate formulations in healthy Korean participants. The mean concentration–time profiles and PK parameters of fenofibric acid were comparable between the test and reference formulations. The GMRs and 90% CIs for the *C*_max_ and AUCs (AUC_last_ and AUC_inf_) of the test formulation relative to the reference formulation were within the bioequivalence limit of 0.80 to 1.25. All AEs were mild except for one moderate AE (nausea) in a participant receiving the reference formulation; no serious AEs occurred during the study period.

The study design, including the number of participants, sampling points, and washout period, was appropriate. The maximum ISV of the primary endpoints (*C*_max_ and AUC_last_) was calculated as 11.0% (*C*_max_) based on the study results. Accordingly, the sample size was calculated to be eight participants, with a statistical significance level of 0.05, considering this ISV. Given that 38 participants completed the study, the sample size was more than adequate to effectively compare the PKs of the two fenofibrate formulations. Based on our findings, the *t*_1/2_ of fenofibric acid was approximately 20 h. Blood samples were collected for up to 72 h post-dose, with a 14-day washout period. These sampling times and washout period exceeded three and five times the *t*_1/2_, respectively, in accordance with the relevant guidelines (Ministry of Food and Drug Safety (KR) 2023).

The GMR and 90% CI for the *C*_max_ of the test formulation relative to the reference formulation was 0.8643 (0.8283–0.9019), while the GMR and 90% CI for the AUC_last_ of the test formulation relative to the reference formulation were 0.9930 (0.9631–1.0239), demonstrating comparable systemic exposure between the test and reference formulations. Although the upper bound of the *C*_max_ CI was below 1.0, the entire CI remained within the bioequivalence limits of 0.80–1.25, confirming regulatory bioequivalence. An ANOVA performed on log-transformed *C*_max_ using a linear mixed-effects model demonstrated a significant treatment effect (*P* < 0.0001), whereas sequence (*P* = 0.2412) and period (*P* = 0.3347) effects were not statistically significant. The slightly lower upper bound of *C*_max_ may be due to ISV or formulation-related differences. However, as AUC_last_ was comparable between the two formulations, the observed *C*_max_ difference is unlikely to be clinically meaningful.

In conclusion, this study demonstrates that the test formulation AD-104 is bioequivalent to the reference formulation TRICOR in healthy Korean participants. Both formulations were safe and well tolerated. Therefore, AD-104 can be reasonably expected to benefit Korean patients with dyslipidemia.

## Supplementary Information

Below is the link to the electronic supplementary material.

Supplementary file1 (DOCX 87.9 KB)

Supplementary file2 (DOCX 21 KB)

## Data Availability

All source data for this work (or generated in this study) are available upon reasonable request.

## References

[CR1] Brown WV (1988) Focus on fenofibrate. Hosp Pract (Off Ed) 23(Suppl 1):31–40. 10.1080/21548331.1988.11703636 3134383 10.1080/21548331.1988.11703636

[CR2] Catapano AL, Graham I, De Backer G, Wiklund O, Chapman MJ, Drexel H, Hoes AW, Jennings CS, Landmesser U, Pedersen TR, Reiner Z, Riccardi G, Taskinen MR, Tokgozoglu L, Monique WM, Verschuren WMM, Vlachopoulos C, Wood DA, Zamorano JL (2016) 2016 ESC/EAS Guidelines for the management of dyslipidaemias. Eur Heart J 37(39):2999. 10.1093/eurheartj/ehw272 27567407 10.1093/eurheartj/ehw272

[CR3] Chow SC, Wang HS (2001) On sample size calculation in bioequivalence trials. J Pharmacokinet Pharmacodyn 28(2):155–169. 10.1023/a:1011503032353 11381568 10.1023/a:1011503032353

[CR4] Collaborators CTT (2013) The effects of lowering LDL cholesterol with statin therapy in people at low risk of vascular disease: meta-analysis of individual data from 27 randomised trials. J Vasc Surg 57(1):284–284 <Go to ISI>://WOS:000312833800056 10.1016/S0140-6736(12)60367-5 PMC3437972 22607822

[CR5] Ginsberg HN, Elam MB, Lovato LC, Crouse JR, Leiter LA, Linz P, Friedewald WT, Buse JB, Gerstein HC, Probstfield J, Grimm RH, Ismail-Beigi F, Bigger JT, Goff DC, Cushman WC, Simons-Morton DG, Byington RP, Grp AS (2010) Effects of combination lipid therapy in type 2 diabetes mellitus. N Engl J Med 362(17):1563–1574. 10.1056/NEJMoa1001282 20228404 10.1056/NEJMoa1001282 PMC2879499

[CR6] Grundy SM, Becker D, Clark LT, Cooper RS, Denke MA, Howard WJ, Hunninghake DB, Illingworth R, Luepker RV, McBride P, McKenney JM, Pasternak RC, Stone NJ, Van Horn L, Brewer HB, Cleeman JI, Ernst ND, Gordon D, Levy D, ... Natl Cholesterol Educ Program E (2002) Third Report of the National Cholesterol Education Program (NCEP) expert panel on detection, evaluation, and treatment of high blood cholesterol in adults (Adult Treatment Panel III) Final Report. Circulation 106(25):3143–3421. 10.1161/circ.106.25.3143

[CR7] Grundy SM, Stone NJ, Bailey AL, Beam C, Birtcher KK, Blumenthal RS, Braun LT, de Ferranti S, Faiella-Tommasino J, Forman DE, Goldberg R, Heidenreich PA, Hlatky MA, Jones DW, Lloyd-Jones D, Lopez-Pajares N, Ndumele CE, Orringer CE, Peralta CA, ... Yeboah J (2019) 2018 AHA/ACC/AACVPR/AAPA/ABC/ACPM/ADA/AGS/APhA/ASPC/NLA/PCNA Guideline on the Management of Blood Cholesterol: Executive Summary: A Report of the American College of Cardiology/American Heart Association Task Force on Clinical Practice Guidelines. Circulation 139(25):E1046-E1081. 10.1161/cir.0000000000000624 10.1161/CIR.0000000000000624 30565953

[CR8] Jones PH, Davidson MH, Goldberg AC, Pepine CJ, Kelly MT, Buttler SM, Setze CM, Lele A, Sleep DJ, Stolzenbach JC (2009) Efficacy and safety of fenofibric acid in combination with a statin in patients with mixed dyslipidemia: pooled analysis of three phase 3, 12-week randomized, controlled studies. J Clin Lipidol 3(2):125–137. 10.1016/j.jacl.2009.02.007 21291802 10.1016/j.jacl.2009.02.007

[CR9] Ministry of Food and Drug Safety (KR) (2023) Guidance document for bioequivalence studies. Ministry of Food and Drug Safety, Cheongju

[CR10] Staels B, Dallongeville J, Auwerx J, Schoonjans K, Leitersdorf E, Fruchart JG (1998) Mechanism of action of fibrates on lipid and lipoprotein metabolism. Circulation 98(19):2088–2093. 10.1161/01.Cir.98.19.2088 9808609 10.1161/01.cir.98.19.2088

[CR11] U.S. Food and Drug Administration (2004) Tricor (fenofibrate) approval documents. https://www.accessdata.fda.gov/drugsatfda_docs/nda/2004/021656s000_TricorTOC.cfm. Accessed 10 Nov 2024

[CR12] U.S. Food and Drug Administration (2018) TRICOR^®^ 48 mg and 145 mg (fenofibrate tablets), Prescribing information. Label approved on 11/2018. https://www.accessdata.fda.gov/drugsatfda_docs/label/2019/021656s029lbl.pdf. Accessed 10 Nov 2024

[CR13] U.S. Food and Drug Administration, Center for Drug Evaluation and Research (2004) Clinical Pharmacology and Biopharmaceutics Review(s): NDA 21–656, TRICOR^®^ (Fenofibric acid). U.S. Food and Drug Administration. https://www.accessdata.fda.gov/drugsatfda_docs/nda/2004/021656s000_Tricor_BioPharmR.pdf. Accessed 14 Feb 2025

[CR14] U.S. Food and Drug Administration, Center for Drug Evaluation and Research (2009) Clinical Pharmacology and Biopharmaceutics Review(s): NDA 22–418, Fibricor™ (Fenofibric acid). U.S. Food and Drug Administration. https://www.accessdata.fda.gov/drugsatfda_docs/nda/2009/022418s000_ClinPharmR.pdf. Accessed 12 Nov 2024

[CR15] World Health Organization (2021) Cardiovascular diseases (CVDs) Fact sheet. Geneva: World Health Organization. https://www.who.int/news-room/fact-sheets/detail/cardiovascular-diseases-(cvds). Accessed 9 Nov 2024

