# Supplementary material for: Comparative pharmacokinetics and bioequivalence of 145-mg fenofibrate formulations in healthy Korean participants
Source: Naunyn Schmiedebergs Arch Pharmacol. 2025 Apr 30;398(10):14461–6. doi: 10.1007/s00210-025-04086-y (PMC12511228; doi:10.1007/s00210-025-04086-y)
Supplement: Supplementary file 1 — Supplementary file1 (DOCX 87.9 KB) [file 210_2025_4086_MOESM1_ESM.docx]

**Comparative Pharmacokinetics and Bioequivalence of 145 mg Fenofibrate Formulations in Healthy Korean participants**

Sujong Lee^1^, Byungwook Kim^1^, SeungHwan Lee^1^, Seung-Hyun Kang^2^, Kyung-Sang Yu^1^

^1^ Department of Clinical Pharmacology and Therapeutics, Seoul National University College of Medicine and Hospital, Seoul, Republic of Korea.

^2^ Clinical Research Center, H-Plus Yangji Hospital, Seoul, Republic of Korea

**Corresponding author:** Kyung-Sang Yu^1^

Department of Clinical Pharmacology and Therapeutics, Seoul National University College of Medicine and Hospital, Seoul, Republic of Korea

Email: [ksyu@snu.ac.kr](mailto:ksyu@snu.ac.kr)

**Validation results of plasma fenofibric acid determination**

No significant interference was detected at the retention time for fenofibric acid and fenofibric aicd-d_6_. The mean within-run accuracy of the quality control (QC) plasma fenofibric acid samples ranged from 91.4 to 96.1%, with imprecision between 2.6 and 2.7% across concentrations ranging from 50.0 ng/mL to 15,000 ng/mL. The mean between-run accuracy ranged from 89.0 to 94.7%, with imprecision between 3.9 and 4.6% over the same concentration range. The percent coefficient of variation (%CV) of the internal standard-normalized matrix effect was 2.6%.

The stock solution stability of fenofibric acid, expressed as percent difference (%D), was 1.9% at room temperature for 6 hours and 0.4% at 5°C for 19 days. For fenofibric aicd-d6, the values were +2.2% and +0.7%, respectively. A summary of the stability study results under various conditions is provided in Supplementary table 1. The QC samples remained stable for 24 hours at room temperature and for 76 days in a deep freezer (-70°C). In an autosampler maintained at 5°C, plasma samples of fenofibric acid were stable for 38 hours. Freeze-thaw stability results showed that fenofibric aicd was stable for at least four freeze-thaw cycles. The mean accuracy and imprecision in the dilution factor were 102.7 and 2.8%, respectively, for a dilution factor of 5. For a dilution factor of 2, the mean accuracy and imprecision were 104.3% and 1.5%, respectively.

Supplementary Table 1. Stability data for plasma fenofibric acid sample

| Concentration (ng/mL) | Short-term stability (at room temperature, 24 hours) | | Stability of the analyte in processed samples (Autosampler stability at 5°C, 38 hours) | | Freeze-thaw stability  (-70°C, 4 cycles) | | Long-term stability  (-70℃, 76 days) | |
| --- | --- | --- | --- | --- | --- | --- | --- | --- |
|  | CV (%) | Difference (%) | CV (%) | Difference (%) | CV (%) | Difference (%) | CV (%) | Difference (%) |
| 150 (QC-Low) | 4.1 | -4.7 | 3.9 | 1.3 | 3.5 | 3.3 | 1.2 | -4.0 |
| 15,000 (QC-High) | 2.9 | -6.7 | 3.2 | 0.0 | 1.9 | -0.7 | 1.0 | -3.3 |
| CV, coefficient of variation; QC, quality control. | | | | | | | | |

Supplementary Table 2. Adverse drug reactions after a single oral administration of the two 145 mg fenofibrate formulations

| Adverse drug reactions | Test  (n = 39) | Reference  (n = 39) | Total  (n = 40) | |
| --- | --- | --- | --- | --- |
| Total | 4 (10.3) [5] | 2 (5.1) [3] | 6 (15.0) [8] | |
| White blood cells urine positive | 2 (5.1) [2] | 0 (0.0) [0] | 2 (5.0) [2] | |
| Blood glucose increased | 1 (2.6) [1] | 0 (0.0) [0] | 1 (2.5) [1] | |
| Haemoglobin decreased | 1 (2.6) [1] | 0 (0.0) [0] | 1 (2.5) [1] | |
| Lipase increased | 0 (0.0) [0] | 1 (2.6) [1] | 1 (2.5) [1] | |
| Neutrophil percentage increased | 0 (0.0) [0] | 1 (2.6) [1] | 1 (2.5) [1] | |
| Red blood cells urine positive | 1 (2.6) [1] | 0 (0.0) [0] | 1 (2.5) [1] | |
| Nausea | 0 (0.0) [0] | 1 (2.6) [1] | 1 (2.5) [1] | |
| Percentages are based on the participants within each treatment group. Data are presented as the number of participants (percentage of participants) [number of events]. | | | |  |


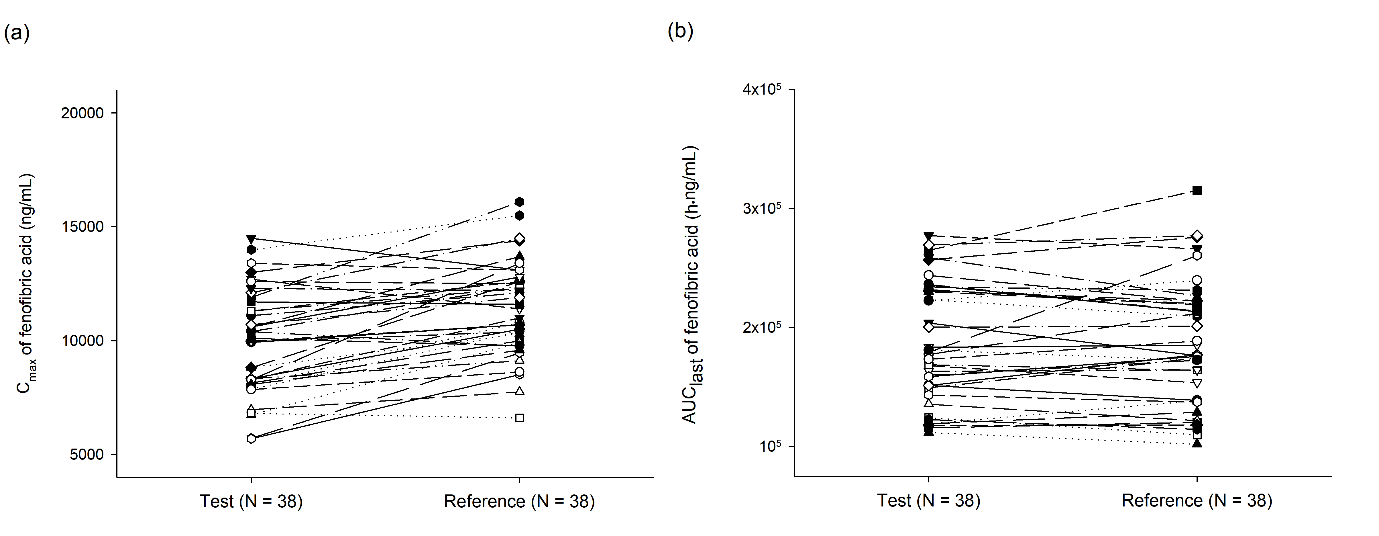


Supplementary Fig. 1 Comparison of (a) C_max_ and (b) AUC_last_ between the test and reference 145 mg fenofibrate formulations in each participant. AUC_last_, area under the concentration-time curve from time zero to the last measurable plasma concentration; C_max_, maximum plasma concentration.
