# Supplementary material for: Comparative pharmacokinetics and bioequivalence of 145-mg fenofibrate formulations in healthy Korean participants
Source: Naunyn Schmiedebergs Arch Pharmacol. 2025 Apr 30;398(10):14461–6. doi: 10.1007/s00210-025-04086-y (PMC12511228; doi:10.1007/s00210-025-04086-y)
Supplement: Supplementary file 2 — Supplementary file2 (DOCX 21 KB) [file 210_2025_4086_MOESM2_ESM.docx]

**Comparative Pharmacokinetics and Bioequivalence of 145 mg Fenofibrate Formulations in Healthy Korean participants**

Sujong Lee^1^, Byungwook Kim^1^, SeungHwan Lee^1^, Seung-Hyun Kang^2^, Kyung-Sang Yu^1^

^1^ Department of Clinical Pharmacology and Therapeutics, Seoul National University College of Medicine and Hospital, Seoul, Republic of Korea.

^2^ Clinical Research Center, H-Plus Yangji Hospital, Seoul, Republic of Korea

**Corresponding author:** Kyung-Sang Yu^1^

Department of Clinical Pharmacology and Therapeutics, Seoul National University College of Medicine and Hospital, Seoul, Republic of Korea

Email: [ksyu@snu.ac.kr](mailto:ksyu@snu.ac.kr)

**Inclusion criteria**

1. A person who is 19 years of age or older at the time of a screening visit

2. A person who has no birth or chronic disease based on screening and has no medical examination results (e.g., electrocardiogram, chest and gastroscopy or gastrointestinal radiography if necessary) or no pathological symptoms or findings

3. A person who is deemed suitable as a test subject as a result of clinical laboratory tests (hematology tests, blood chemistry tests, urine tests, etc.) established and conducted by a person in charge of testing (or a delegated doctor in charge of testing) according to the characteristics of investigational product

4. Those with the following blood pressure

- Systolic blood pressure not less than 90 mmHg but not more than 139 mmHg
- The diastolic blood pressure is greater than or equal to 60 mmHg and less than or equal to 89 mmHg

5. Those whose BMI measurement results are greater than or equal to 18 kg/m^2^ and less than or equal to 30 kg/m^2^ at the time of a screening visit

- BMI (kg/m^2^) = Weight (kg) / Height (m)^2^

6. Those who have no history of gastrointestinal diseases or gastrointestinal resection that can affect the absorption of clinical trial drugs

7. A person who has no history of mental illness within five years prior to the date of the screening visit

8. In the case of a female test subject, a person who is confirmed not to be pregnant at the time of a medical examination

9. A person who has signed a consent form according to his/her free will after hearing and understanding the purpose, contents, characteristics of the drug, expected adverse reactions, etc. of this test

**Exclusion criteria**

1. A person who has taken drug metabolite-inducing and inhibiting drugs such as barbiturant drugs within one month before the start of the test (the first administration date)

2. Drug users who are likely to interfere with testing within 10 days before the start of the test (the first day of administration)

3. Excessive alcohol drinkers within one month prior to the date of the screening visit

- More than 14 cups/week for women
- More than 21 cups/week for men

(1 glass = 50mL of soju or 30mL of spirits or 250mL of beer)

4. A person who has participated in other clinical trials or biological equivalence trials within six months prior to the implementation of the relevant test (the date of first administration)

5. A person who has donated whole blood or donated ingredients within two weeks before the start of the test (the first date of administration), or received a blood transfusion within one month

6. A person who is not qualified for participation in this clinical trial by a person in charge of the trial (or a delegated test doctor)

7. Those whose blood AST (GOT) or ALT (GPT) levels exceed twice the upper limit of the reference range or whose γ-GTP levels exceed 1.5 times the upper limit of the reference range

8. A person with a liver disorder

9. People with moderate to severe renal impairment (serum creatinine level greater than 2.5 mg/dL)

10. A person who is hypersensitive to this drug and its components

11. Those with pre-existing gallbladder disease

12. Those who have experienced photoallergy or phototoxicity during treatment with fibrate or ketoprofen

13. A person with bile duct cirrhosis

14. Those with pancreatitis (excluding acute pancreatitis caused by severe hypertriglyceridemia)

15. A lactating women

16. A person who does not agree to medically exclude the possibility of pregnancy by using contraception* by himself, his/her spouse, or his/her partner from the date of first administration of the clinical trial drug until 14 days after the date of last administration of the clinical trial drug

* Contraception: Use a combination of intrauterine devices (IUD, IUS), vasectomy, oviduct ligation and blocking contraception (male condom, female condom, cervical cap, contraceptive septum, sponge, etc.), or use a combination of two or more blocking contraception when using a fertilizer
